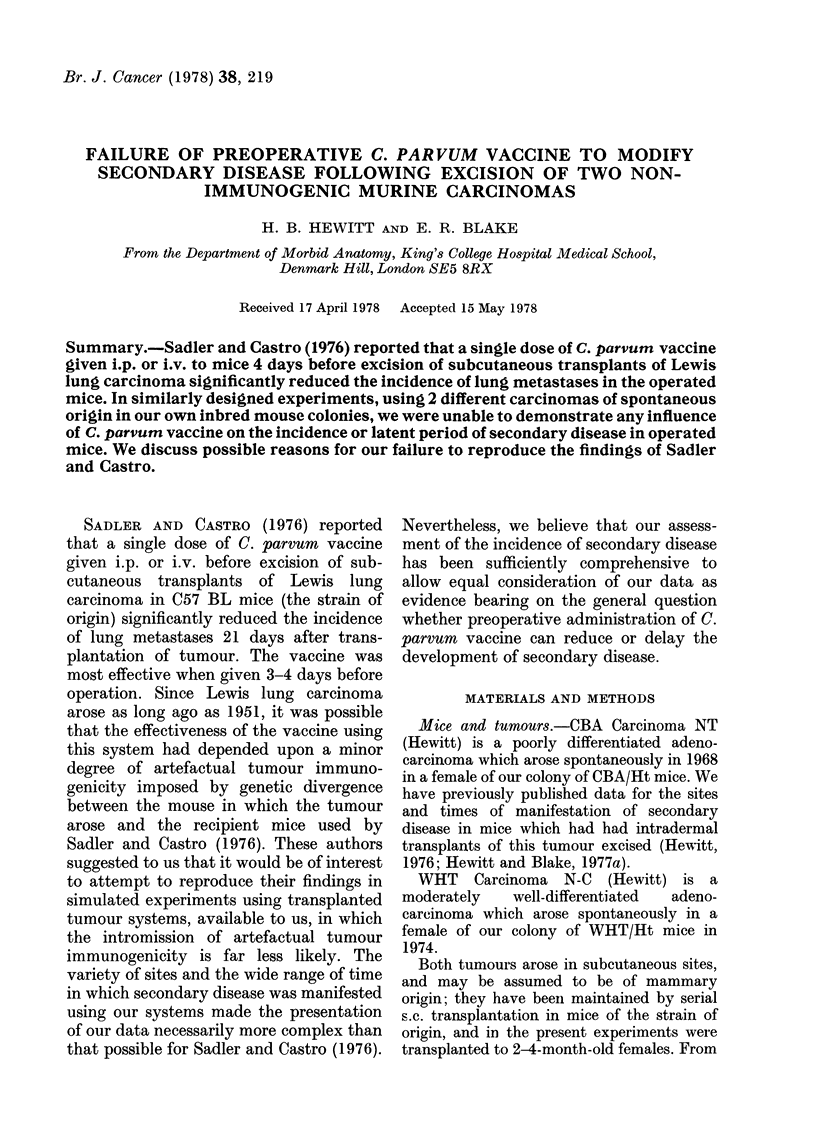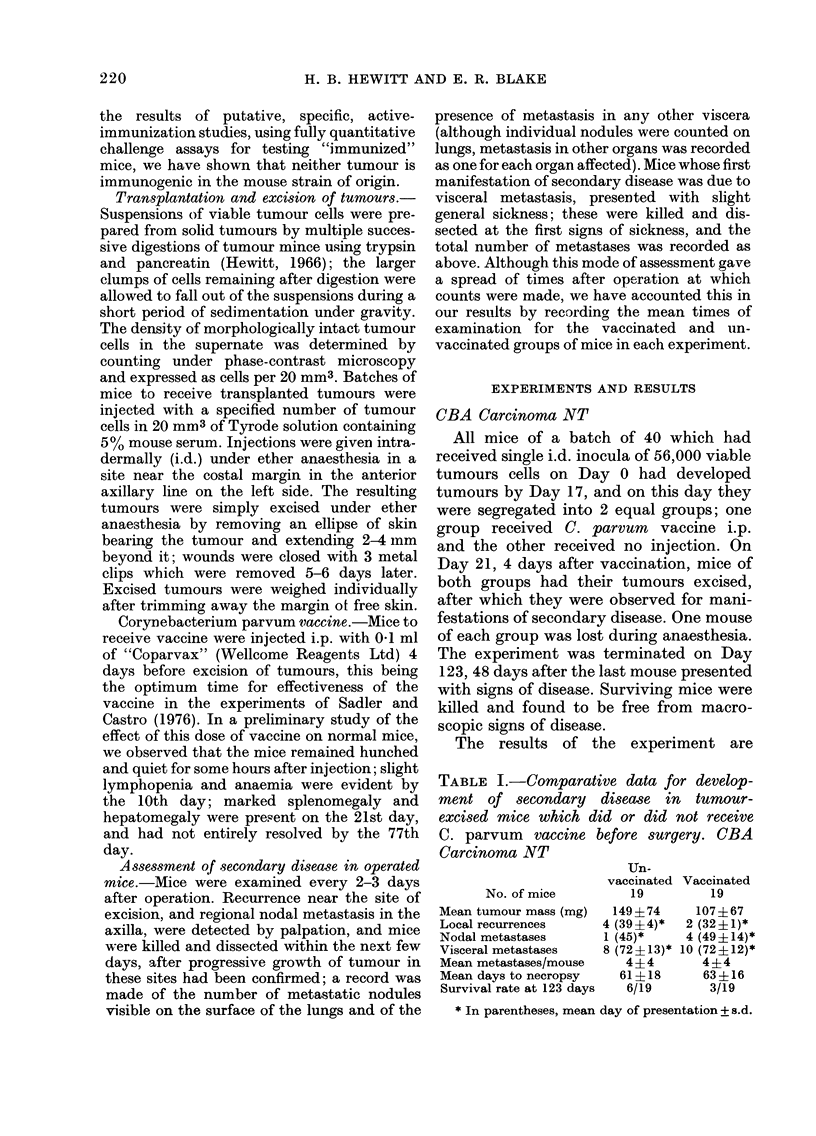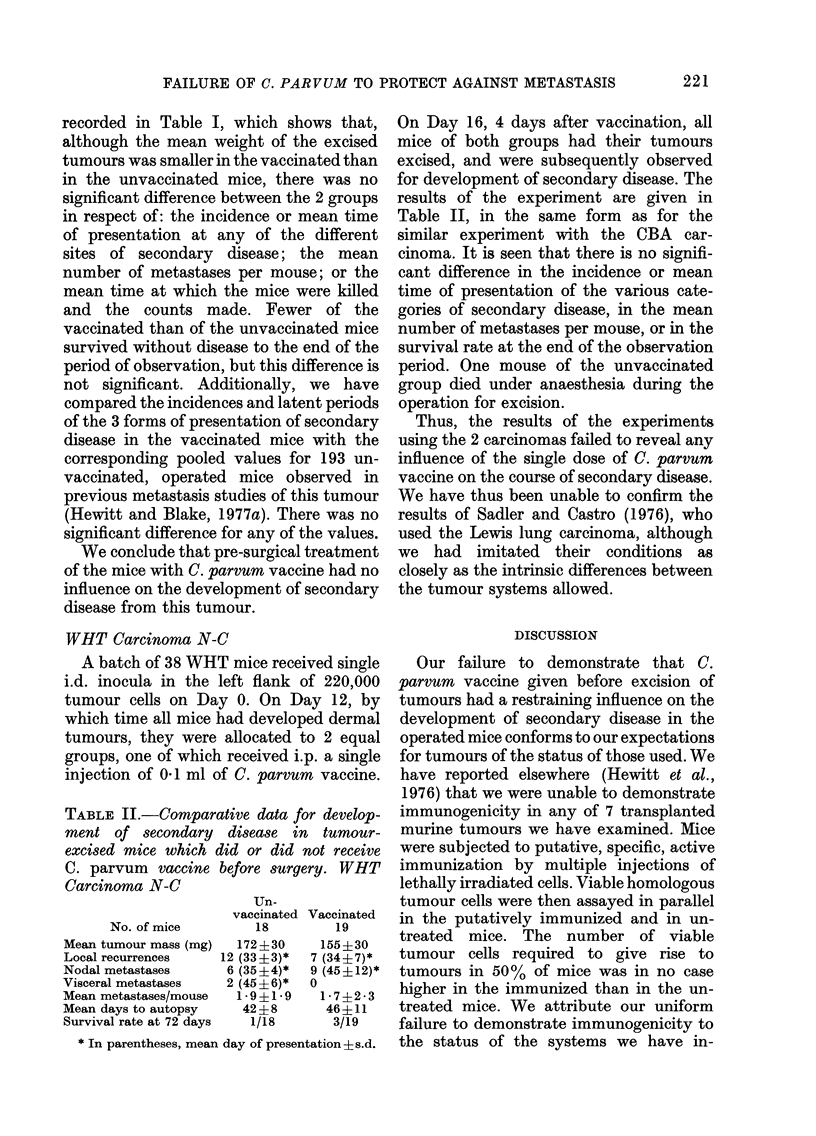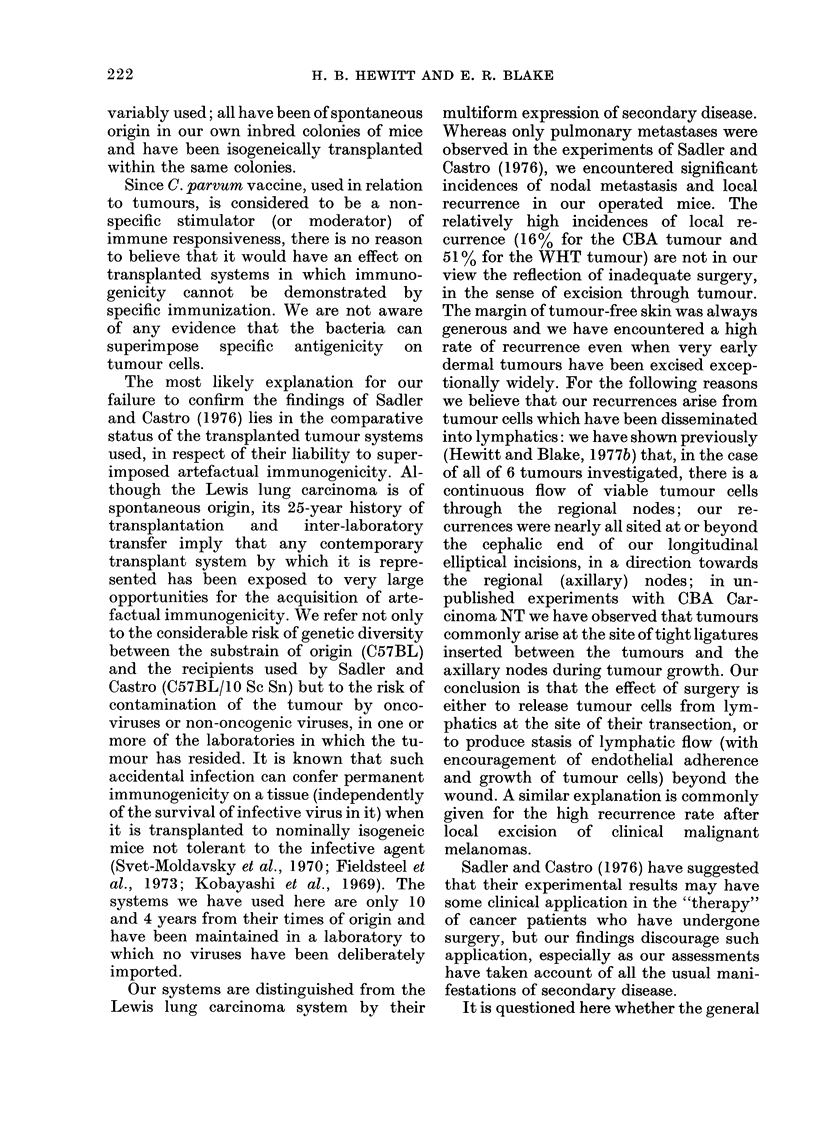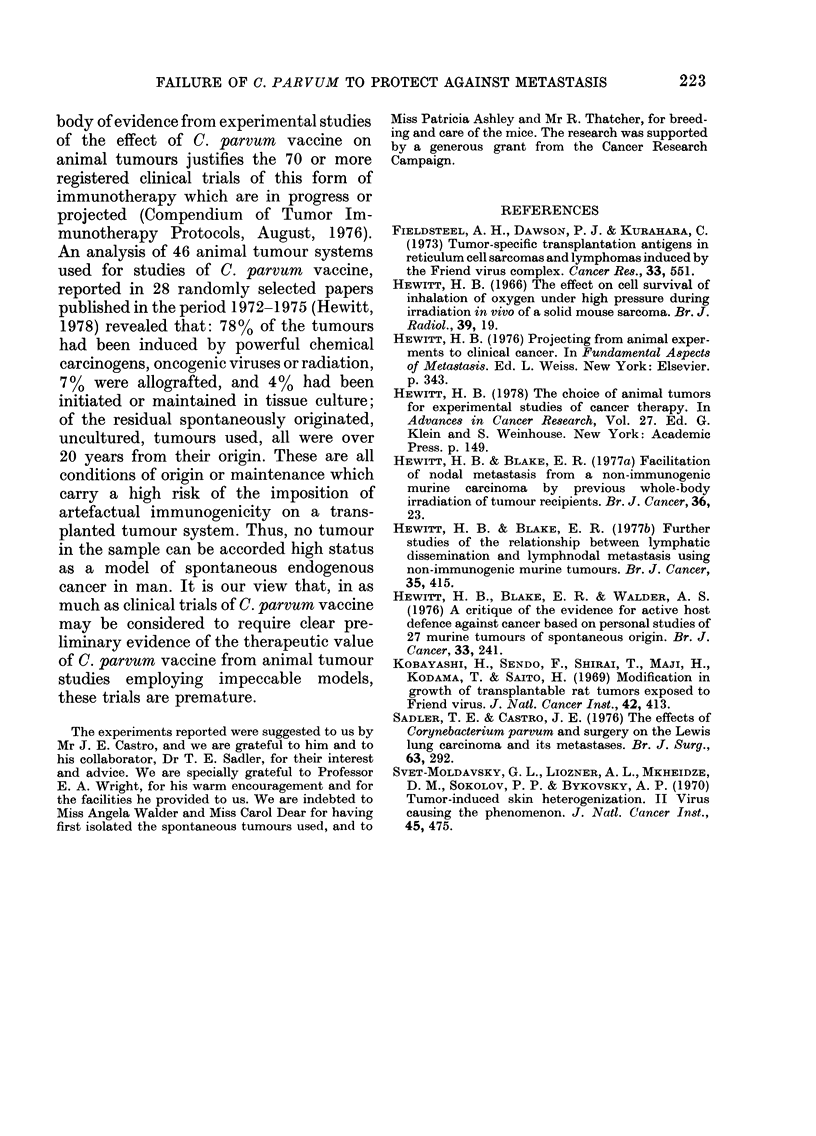# Failure of preoperative C. parvum vaccine to modify secondary disease following excision of two non-immunogenic murine carcinomas.

**DOI:** 10.1038/bjc.1978.191

**Published:** 1978-08

**Authors:** H. B. Hewitt, E. R. Blake

## Abstract

Sadler and Castro (1976) reported that a single dose of C. parvum vaccine given i.p. or i.v. to mice 4 days before excision of subcutaneous transplants of Lewis lung carcinoma significantly reduced the incidence of lung metastases in the operated mice. In similarly designed experiments, using 2 different carcinomas of spontaneous origin in our own inbred mouse colonies, we were unable to demonstrate any influence of C. parvum vaccine on the incidence or latent period of secondary disease in operated mice. We discuss possible reasons for our failure to reproduce the findings of Sadler and Castro.


*Br. J. Cancer* (1978) **38**, 219

### FAILURE OF PREOPERATIVE *C. PARVUM* VACCINE TO MODIFY SECONDARY DISEASE FOLLOWING EXCISION OF TWO NON-IMMUNOGENIC MURINE CARCINOMAS

H. B. HEWITT AND E. R. BLAKE

*From the Department of Morbid Anatomy, King's College Hospital Medical School, Denmark Hill, London SE5 8RX*

Received 17 April 1978 Accepted 15 May 1978

**Summary.—Sadler and Castro (1976) reported that a single dose of *C. parvum* vaccine given i.p. or i.v. to mice 4 days before excision of subcutaneous transplants of Lewis lung carcinoma significantly reduced the incidence of lung metastases in the operated mice. In similarly designed experiments, using 2 different carcinomas of spontaneous origin in our own inbred mouse colonies, we were unable to demonstrate any influence of *C. parvum* vaccine on the incidence or latent period of secondary disease in operated mice. We discuss possible reasons for our failure to reproduce the findings of Sadler and Castro.**

Sadler and Castro (1976) reported that a single dose of *C. parvum* vaccine given i.p. or i.v. before excision of subcutaneous transplants of Lewis lung carcinoma in C57 BL mice (the strain of origin) significantly reduced the incidence of lung metastases 21 days after transplantation of tumour. The vaccine was most effective when given 3–4 days before operation. Since Lewis lung carcinoma arose as long ago as 1951, it was possible that the effectiveness of the vaccine using this system had depended upon a minor degree of artefactual tumour immunogenicity imposed by genetic divergence between the mouse in which the tumour arose and the recipient mice used by Sadler and Castro (1976). These authors suggested to us that it would be of interest to attempt to reproduce their findings in simulated experiments using transplanted tumour systems, available to us, in which the intromission of artefactual tumour immunogenicity is far less likely. The variety of sites and the wide range of time in which secondary disease was manifested using our systems made the presentation of our data necessarily more complex than that possible for Sadler and Castro (1976). Nevertheless, we believe that our assessment of the incidence of secondary disease has been sufficiently comprehensive to allow equal consideration of our data as evidence bearing on the general question whether preoperative administration of *C. parvum* vaccine can reduce or delay the development of secondary disease.

### MATERIALS AND METHODS

*Mice and tumours.*—CBA Carcinoma NT (Hewitt) is a poorly differentiated adenocarcinoma which arose spontaneously in 1968 in a female of our colony of CBA/Ht mice. We have previously published data for the sites and times of manifestation of secondary disease in mice which had had intradermal transplants of this tumour excised (Hewitt, 1976; Hewitt and Blake, 1977*a*).

WHT Carcinoma N-C (Hewitt) is a moderately well-differentiated adenocarcinoma which arose spontaneously in a female of our colony of WHT/Ht mice in 1974.

Both tumours arose in subcutaneous sites, and may be assumed to be of mammary origin; they have been maintained by serial s.c. transplantation in mice of the strain of origin, and in the present experiments were transplanted to 2–4-month-old females. From the results of putative, specific, active-immunization studies, using fully quantitative challenge assays for testing "immunized" mice, we have shown that neither tumour is immunogenic in the mouse strain of origin.

*Transplantation and excision of tumours.*—Suspensions of viable tumour cells were prepared from solid tumours by multiple successive digestions of tumour mince using trypsin and pancreatin (Hewitt, 1966); the larger clumps of cells remaining after digestion were allowed to fall out of the suspensions during a short period of sedimentation under gravity. The density of morphologically intact tumour cells in the supernate was determined by counting under phase-contrast microscopy and expressed as cells per 20 $mm^3$. Batches of mice to receive transplanted tumours were injected with a specified number of tumour cells in 20 $mm^3$ of Tyrode solution containing 5% mouse serum. Injections were given intradermally (i.d.) under ether anaesthesia in a site near the costal margin in the anterior axillary line on the left side. The resulting tumours were simply excised under ether anaesthesia by removing an ellipse of skin bearing the tumour and extending 2–4 mm beyond it; wounds were closed with 3 metal clips which were removed 5–6 days later. Excised tumours were weighed individually after trimming away the margin of free skin.

Corynebacterium parvum *vaccine.*—Mice to receive vaccine were injected i.p. with 0·1 ml of "Coparvax" (Wellcome Reagents Ltd) 4 days before excision of tumours, this being the optimum time for effectiveness of the vaccine in the experiments of Sadler and Castro (1976). In a preliminary study of the effect of this dose of vaccine on normal mice, we observed that the mice remained hunched and quiet for some hours after injection; slight lymphopenia and anaemia were evident by the 10th day; marked splenomegaly and hepatomegaly were present on the 21st day, and had not entirely resolved by the 77th day.

*Assessment of secondary disease in operated mice.*—Mice were examined every 2–3 days after operation. Recurrence near the site of excision, and regional nodal metastasis in the axilla, were detected by palpation, and mice were killed and dissected within the next few days, after progressive growth of tumour in these sites had been confirmed; a record was made of the number of metastatic nodules visible on the surface of the lungs and of the presence of metastasis in any other viscera (although individual nodules were counted on lungs, metastasis in other organs was recorded as one for each organ affected). Mice whose first manifestation of secondary disease was due to visceral metastasis, presented with slight general sickness; these were killed and dissected at the first signs of sickness, and the total number of metastases was recorded as above. Although this mode of assessment gave a spread of times after operation at which counts were made, we have accounted this in our results by recording the mean times of examination for the vaccinated and unvaccinated groups of mice in each experiment.

### EXPERIMENTS AND RESULTS

### *CBA Carcinoma NT*

All mice of a batch of 40 which had received single i.d. inocula of 56,000 viable tumours cells on Day 0 had developed tumours by Day 17, and on this day they were segregated into 2 equal groups; one group received *C. parvum* vaccine i.p. and the other received no injection. On Day 21, 4 days after vaccination, mice of both groups had their tumours excised, after which they were observed for manifestations of secondary disease. One mouse of each group was lost during anaesthesia. The experiment was terminated on Day 123, 48 days after the last mouse presented with signs of disease. Surviving mice were killed and found to be free from macroscopic signs of disease.

The results of the experiment are

TABLE I.—*Comparative data for development of secondary disease in tumour-excised mice which did or did not receive* C. parvum *vaccine before surgery. CBA Carcinoma NT*

| | Un-vaccinated | Vaccinated |
|---|---|---|
| No. of mice | 19 | 19 |
| Mean tumour mass (mg) | 149 ± 74 | 107 ± 67 |
| Local recurrences | 4 (39 ± 4)* | 2 (32 ± 1)* |
| Nodal metastases | 1 (45)* | 4 (49 ± 14)* |
| Visceral metastases | 8 (72 ± 13)* | 10 (72 ± 12)* |
| Mean metastases/mouse | 4 ± 4 | 4 ± 4 |
| Mean days to necropsy | 61 ± 18 | 63 ± 16 |
| Survival rate at 123 days | 6/19 | 3/19 |

* In parentheses, mean day of presentation ± s.d.

recorded in Table I, which shows that, although the mean weight of the excised tumours was smaller in the vaccinated than in the unvaccinated mice, there was no significant difference between the 2 groups in respect of: the incidence or mean time of presentation at any of the different sites of secondary disease; the mean number of metastases per mouse; or the mean time at which the mice were killed and the counts made. Fewer of the vaccinated than of the unvaccinated mice survived without disease to the end of the period of observation, but this difference is not significant. Additionally, we have compared the incidences and latent periods of the 3 forms of presentation of secondary disease in the vaccinated mice with the corresponding pooled values for 193 unvaccinated, operated mice observed in previous metastasis studies of this tumour (Hewitt and Blake, 1977*a*). There was no significant difference for any of the values.

We conclude that pre-surgical treatment of the mice with *C. parvum* vaccine had no influence on the development of secondary disease from this tumour.

### *WHT Carcinoma N-C*

A batch of 38 WHT mice received single i.d. inocula in the left flank of 220,000 tumour cells on Day 0. On Day 12, by which time all mice had developed dermal tumours, they were allocated to 2 equal groups, one of which received i.p. a single injection of 0·1 ml of *C. parvum* vaccine.

TABLE II.—*Comparative data for development of secondary disease in tumour-excised mice which did or did not receive* C. parvum *vaccine before surgery. WHT Carcinoma N-C*

| No. of mice | Unvaccinated 18 | Vaccinated 19 |
|---|---|---|
| Mean tumour mass (mg) | 172±30 | 155±30 |
| Local recurrences | 12 (33±3)* | 7 (34±7)* |
| Nodal metastases | 6 (35±4)* | 9 (45±12)* |
| Visceral metastases | 2 (45±6)* | 0 |
| Mean metastases/mouse | 1·9±1·9 | 1·7±2·3 |
| Mean days to autopsy | 42±8 | 46±11 |
| Survival rate at 72 days | 1/18 | 3/19 |

* In parentheses, mean day of presentation ±s.d.

On Day 16, 4 days after vaccination, all mice of both groups had their tumours excised, and were subsequently observed for development of secondary disease. The results of the experiment are given in Table II, in the same form as for the similar experiment with the CBA carcinoma. It is seen that there is no significant difference in the incidence or mean time of presentation of the various categories of secondary disease, in the mean number of metastases per mouse, or in the survival rate at the end of the observation period. One mouse of the unvaccinated group died under anaesthesia during the operation for excision.

Thus, the results of the experiments using the 2 carcinomas failed to reveal any influence of the single dose of *C. parvum* vaccine on the course of secondary disease. We have thus been unable to confirm the results of Sadler and Castro (1976), who used the Lewis lung carcinoma, although we had imitated their conditions as closely as the intrinsic differences between the tumour systems allowed.

### DISCUSSION

Our failure to demonstrate that *C. parvum* vaccine given before excision of tumours had a restraining influence on the development of secondary disease in the operated mice conforms to our expectations for tumours of the status of those used. We have reported elsewhere (Hewitt *et al.*, 1976) that we were unable to demonstrate immunogenicity in any of 7 transplanted murine tumours we have examined. Mice were subjected to putative, specific, active immunization by multiple injections of lethally irradiated cells. Viable homologous tumour cells were then assayed in parallel in the putatively immunized and in untreated mice. The number of viable tumour cells required to give rise to tumours in 50% of mice was in no case higher in the immunized than in the untreated mice. We attribute our uniform failure to demonstrate immunogenicity to the status of the systems we have invariably used; all have been of spontaneous origin in our own inbred colonies of mice and have been isogeneically transplanted within the same colonies.

Since *C. parvum* vaccine, used in relation to tumours, is considered to be a non-specific stimulator (or moderator) of immune responsiveness, there is no reason to believe that it would have an effect on transplanted systems in which immunogenicity cannot be demonstrated by specific immunization. We are not aware of any evidence that the bacteria can superimpose specific antigenicity on tumour cells.

The most likely explanation for our failure to confirm the findings of Sadler and Castro (1976) lies in the comparative status of the transplanted tumour systems used, in respect of their liability to superimposed artefactual immunogenicity. Although the Lewis lung carcinoma is of spontaneous origin, its 25-year history of transplantation and inter-laboratory transfer imply that any contemporary transplant system by which it is represented has been exposed to very large opportunities for the acquisition of artefactual immunogenicity. We refer not only to the considerable risk of genetic diversity between the substrain of origin (C57BL) and the recipients used by Sadler and Castro (C57BL/10 Sc Sn) but to the risk of contamination of the tumour by oncoviruses or non-oncogenic viruses, in one or more of the laboratories in which the tumour has resided. It is known that such accidental infection can confer permanent immunogenicity on a tissue (independently of the survival of infective virus in it) when it is transplanted to nominally isogeneic mice not tolerant to the infective agent (Svet-Moldavsky *et al.*, 1970; Fieldsteel *et al.*, 1973; Kobayashi *et al.*, 1969). The systems we have used here are only 10 and 4 years from their times of origin and have been maintained in a laboratory to which no viruses have been deliberately imported.

Our systems are distinguished from the Lewis lung carcinoma system by their multiform expression of secondary disease. Whereas only pulmonary metastases were observed in the experiments of Sadler and Castro (1976), we encountered significant incidences of nodal metastasis and local recurrence in our operated mice. The relatively high incidences of local recurrence (16% for the CBA tumour and 51% for the WHT tumour) are not in our view the reflection of inadequate surgery, in the sense of excision through tumour. The margin of tumour-free skin was always generous and we have encountered a high rate of recurrence even when very early dermal tumours have been excised exceptionally widely. For the following reasons we believe that our recurrences arise from tumour cells which have been disseminated into lymphatics: we have shown previously (Hewitt and Blake, 1977*b*) that, in the case of all of 6 tumours investigated, there is a continuous flow of viable tumour cells through the regional nodes; our recurrences were nearly all sited at or beyond the cephalic end of our longitudinal elliptical incisions, in a direction towards the regional (axillary) nodes; in unpublished experiments with CBA Carcinoma NT we have observed that tumours commonly arise at the site of tight ligatures inserted between the tumours and the axillary nodes during tumour growth. Our conclusion is that the effect of surgery is either to release tumour cells from lymphatics at the site of their transection, or to produce stasis of lymphatic flow (with encouragement of endothelial adherence and growth of tumour cells) beyond the wound. A similar explanation is commonly given for the high recurrence rate after local excision of clinical malignant melanomas.

Sadler and Castro (1976) have suggested that their experimental results may have some clinical application in the "therapy" of cancer patients who have undergone surgery, but our findings discourage such application, especially as our assessments have taken account of all the usual manifestations of secondary disease.

It is questioned here whether the general body of evidence from experimental studies of the effect of *C. parvum* vaccine on animal tumours justifies the 70 or more registered clinical trials of this form of immunotherapy which are in progress or projected (Compendium of Tumor Immunotherapy Protocols, August, 1976). An analysis of 46 animal tumour systems used for studies of *C. parvum* vaccine, reported in 28 randomly selected papers published in the period 1972–1975 (Hewitt, 1978) revealed that: 78% of the tumours had been induced by powerful chemical carcinogens, oncogenic viruses or radiation, 7% were allografted, and 4% had been initiated or maintained in tissue culture; of the residual spontaneously originated, uncultured, tumours used, all were over 20 years from their origin. These are all conditions of origin or maintenance which carry a high risk of the imposition of artefactual immunogenicity on a transplanted tumour system. Thus, no tumour in the sample can be accorded high status as a model of spontaneous endogenous cancer in man. It is our view that, in as much as clinical trials of *C. parvum* vaccine may be considered to require clear preliminary evidence of the therapeutic value of *C. parvum* vaccine from animal tumour studies employing impeccable models, these trials are premature.

The experiments reported were suggested to us by Mr J. E. Castro, and we are grateful to him and to his collaborator, Dr T. E. Sadler, for their interest and advice. We are specially grateful to Professor E. A. Wright, for his warm encouragement and for the facilities he provided to us. We are indebted to Miss Angela Walder and Miss Carol Dear for having first isolated the spontaneous tumours used, and to Miss Patricia Ashley and Mr R. Thatcher, for breeding and care of the mice. The research was supported by a generous grant from the Cancer Research Campaign.

### REFERENCES

FIELDSTEEL, A. H., DAWSON, P. J. & KURAHARA, C. (1973) Tumor-specific transplantation antigens in reticulum cell sarcomas and lymphomas induced by the Friend virus complex. *Cancer Res.*, **33**, 551.

HEWITT, H. B. (1966) The effect on cell survival of inhalation of oxygen under high pressure during irradiation *in vivo* of a solid mouse sarcoma. *Br. J. Radiol.*, **39**, 19.

HEWITT, H. B. (1976) Projecting from animal experments to clinical cancer. In *Fundamental Aspects of Metastasis*. Ed. L. Weiss. New York: Elsevier. p. 343.

HEWITT, H. B. (1978) The choice of animal tumors for experimental studies of cancer therapy. In *Advances in Cancer Research*, Vol. 27. Ed. G. Klein and S. Weinhouse. New York: Academic Press. p. 149.

HEWITT, H. B. & BLAKE, E. R. (1977*a*) Facilitation of nodal metastasis from a non-immunogenic murine carcinoma by previous whole-body irradiation of tumour recipients. *Br. J. Cancer*, **36**, 23.

HEWITT, H. B. & BLAKE, E. R. (1977*b*) Further studies of the relationship between lymphatic dissemination and lymphnodal metastasis using non-immunogenic murine tumours. *Br. J. Cancer*, **35**, 415.

HEWITT, H. B., BLAKE, E. R. & WALDER, A. S. (1976) A critique of the evidence for active host defence against cancer based on personal studies of 27 murine tumours of spontaneous origin. *Br. J. Cancer*, **33**, 241.

KOBAYASHI, H., SENDO, F., SHIRAI, T., MAJI, H., KODAMA, T. & SAITO, H. (1969) Modification in growth of transplantable rat tumors exposed to Friend virus. *J. Natl. Cancer Inst.*, **42**, 413.

SADLER, T. E. & CASTRO, J. E. (1976) The effects of *Corynebacterium parvum* and surgery on the Lewis lung carcinoma and its metastases. *Br. J. Surg.*, **63**, 292.

SVET-MOLDAVSKY, G. L., LIOZNER, A. L., MKHEIDZE, D. M., SOKOLOV, P. P. & BYKOVSKY, A. P. (1970) Tumor-induced skin heterogenization. II Virus causing the phenomenon. *J. Natl. Cancer Inst.*, **45**, 475.